# Linking toxicant physiological mode of action with induced gene expression changes in *Caenorhabditis elegans*

**DOI:** 10.1186/1752-0509-4-32

**Published:** 2010-03-23

**Authors:** Suresh Swain, Jodie F Wren, Stephen R Stürzenbaum, Peter Kille, A John Morgan, Tjalling Jager, Martijs J Jonker, Peter K Hankard, Claus Svendsen, Jenifer Owen, B Ann Hedley, Mark Blaxter, David J Spurgeon

**Affiliations:** 1 King's College London, Department of Biochemistry, Pharmaceutical Sciences Research Division, 150 Stamford Street, London SE1 9NH, UK; 2 Centre for Ecology and Hydrology, Maclean Building, Benson Lane, Crowmarsh Gifford, Wallingford, Oxon, OX10 8BB, UK; 3 Cardiff School of Biosciences, BIOSI 1, University of Cardiff, PO Box 915, Cardiff, CF10 3TL, UK; 4 Department of Theoretical Biology, Vrije Universiteit Amsterdam, De Boelelaan 1085, NL-1081 HV Amsterdam, The Netherlands; 5 Microarray Department and Integrative Bioinformatics Unit, Faculty of Science, University of Amsterdam, Kruislaan 318, Building I, Room 105C, 1098 SM Amsterdam, The Netherlands; 6 Integrated Centre for Applied Population Biology, University of Edinburgh, Kings Buildings, Edinburgh, EH9 3JT, UK

## Abstract

**Background:**

Physiologically based modelling using DEBtox (dynamic energy budget in toxicology) and transcriptional profiling were used in *Caenorhabditis elegans *to identify how physiological modes of action, as indicated by effects on system level resource allocation were associated with changes in gene expression following exposure to three toxic chemicals: cadmium, fluoranthene (FA) and atrazine (AZ).

**Results:**

For Cd, the physiological mode of action as indicated by DEBtox model fitting was an effect on energy assimilation from food, suggesting that the transcriptional response to exposure should be dominated by changes in the expression of transcripts associated with energy metabolism and the mitochondria. While evidence for effect on genes associated with energy production were seen, an ontological analysis also indicated an effect of Cd exposure on DNA integrity and transcriptional activity. DEBtox modelling showed an effect of FA on costs for growth and reproduction (i.e. for production of new and differentiated biomass). The microarray analysis supported this effect, showing an effect of FA on protein integrity and turnover that would be expected to have consequences for rates of somatic growth. For AZ, the physiological mode of action predicted by DEBtox was increased cost for maintenance. The transcriptional analysis demonstrated that this increase resulted from effects on DNA integrity as indicated by changes in the expression of genes chromosomal repair.

**Conclusions:**

Our results have established that outputs from process based models and transcriptomics analyses can help to link mechanisms of action of toxic chemicals with resulting demographic effects. Such complimentary analyses can assist in the categorisation of chemicals for risk assessment purposes.

## Background

Organisms display physiologies and life-histories that are determined by a set of interacting biochemical networks [1-4]. These in turn are controlled through regulation of the expression of proteins translated from RNA that itself is ultimately transcribed from the genome. Understanding how gene/protein networks control complex traits and their responses to external cues is one of the great challenges in biology [5-8]. Within this discipline of systems biology, emphasis is currently on coupling bottom-up approaches such as qualitative and quantitative detection of DNA/RNA, proteins and metabolites with data mining and visualisation approaches, thereby allowing elucidation of the networks that control complex biological processes [9-12]. The hypotheses that are generated through this approach can then be validated by focussed studies using loss or gain of function mutants and/or RNAi.

Whilst bottom-up techniques can aid understanding of how gene and protein networks control complex traits, such as those associated with life-history, we are far from fully elucidating the mechanisms through which life-history phenotypes change in response to environmental stimuli. In the case of environmental stress (e.g. toxic chemical exposure), an organism will typically respond by upregulation of a network of protective (e.g. detoxification) and compensatory (e.g. stress proteins) mechanisms [13]. These adaptive changes can alter energy allocation between biological processes, which in conjunction with direct toxic effects, may result in changes in the rate and timing of life-history events.

In physiological ecology, it is well known that life-history traits are not independent but instead are tightly linked as described through rules of metabolic organisation. As an example, a change in feeding rate will have very specific consequences for growth, development and reproduction because organisms are subject to the law of conservation of mass and energy. To understand the basis of such effects, theoretical ecologists have sought to develop top-down models that use phenotypic data to provide an understanding of the physiological mode of action of stress effects.

One of the most prominent ecophysiological systems for modelling the inter-relational links between life-history traits is based on dynamic energy budget (DEB) theory [14,15]. DEB theory explains how organisms acquire energy and allocate resources to key physiological processes such as maintenance, growth and reproduction based on a set of rules for metabolic organisation. The physiological processes that are modelled within the DEB framework all have a clear physiological basis. For example, animals obtain energy from food via the oxidation and reduction reactions of glycolysis, the Krebs cycle, and protein and fatty acid metabolism. Somatic maintenance, i.e. tissue and cell regeneration and recovery from injury, involves stress responses such as protection against oxidative stress, protein chaperoning and DNA repair. Somatic growth occurs through cell division, while reproduction generally requires gamete and egg production. In this way, ecophysiological models such as DEB can provide a top-down approach to predict toxicant impacts on the physiology of individuals that when combined with the bottom-up molecular approach can enhance our knowledge of systemic responses to environmental stress [16-18].

Since its inception, DEB theory has been used to describe how toxicants can alter energy usage in an attempt to bridge the gap between individual and ecosystem-based approaches to toxicology [16]. By examining responses of individuals to chemical exposure in the context of their energy balance, DEB theory can be used to explain how chemical toxicokinetics affects the allocation of energy resources; the extent of associated cellular damage and ultimately the performance of a range of life-history traits [17]. DEBtox can be used to derive a number of parameters including the no effect concentration (NEC), parameters describing chemical kinetics and a killing rate that is linked to internal concentrations [17,19]. All of which provide useful information about mechanisms of effect.

**Figure 1:**
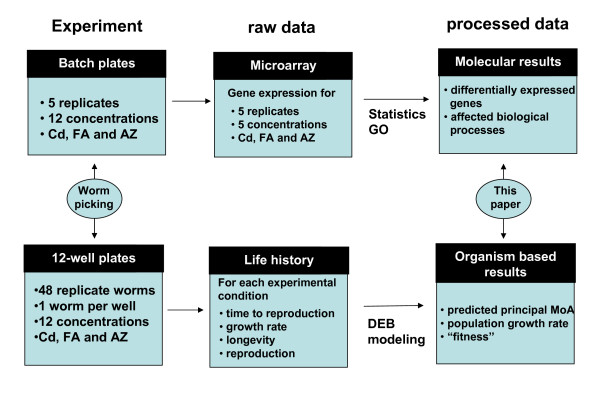
**Experimental design**. Schematic of the experimental design and approaches used for collection of life-history and transcriptional data.

Here we use DEB theory and explicitly a recently developed version of the DEBtox model [17,19], to investigate the physiological mode of action of three different types of chemical stressors (the metal cadmium, the non-polar organic fluoranthene, and the herbicide atrazine) during a whole life-cycle exposure of the nematode *Caenorhabditis elegans*. The three chemicals were selected as representatives of major pollutant sub-groups: cadmium is teratogenic, mutagenic and potentially carcinogenic metals; fluoranthene a non-polar polycyclic aromatic hydrocarbon (PAH) which can be expected to show mainly narcotic toxicity and atrazine a herbicide that is known to inhibit electron transport. Transcriptional profiling is then used to identify the changes in gene expression associated with toxicity that underlie observed changes in life-history traits (experiments designed according to the scheme shown in Fig. 1). The aim was to associate gene expression change for each chemical with the physiological mode of action as indicated by DEBtox modelling. Thus, a comprehensive picture of responses to exposure from transcriptional remodelling through to life-history change in worms exposed to different types of chemical stress was sought.

## Results

### Life-history responses

**Figure 2:**
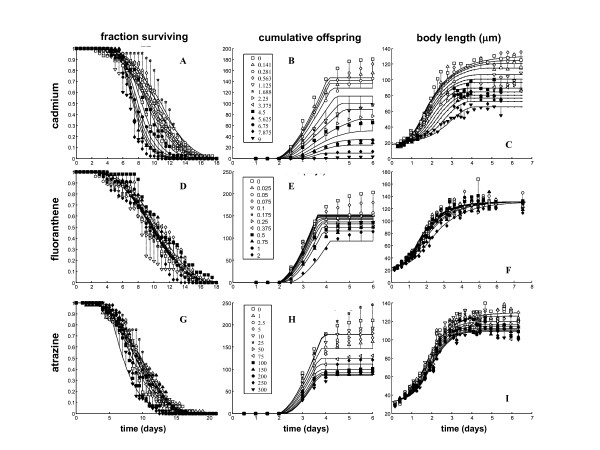
**Life history data and DEBtox fits**. Life-history data and DEBtox fits. Data and simulation model fits for survival (A, D, G), cumulative offspring (B, E, H) and growth (C, F, I) from the life-history toxicity studies with the nematode *C. elegans *conducted for Cd (A, B, C), FA (D, E, F) and AZ (G, H, I).

Comparable values for mean lifespan of control worms (9.0, 8.8 and 9.7 days for Cd, FA and AZ) and control brood size (203, 204 and 211 eggs/worm for Cd, FA and AZ) were found between the three experiments. For Cd, DEBtox fits indicated that exposure affected survival patterns in time (Fig. 2A). Worms exposed to low Cd concentrations showed an increase in lifespan (significant at 1.69 mg/L, Tukey's post-hoc, p < 0.001), although a non-significant reduction was found at high concentrations. DEBtox indicated an effect of Cd on temporal patterns and rates of egg production (Fig. 2, Panel B) and growth (Fig. 2, Panel C). Total broodsize at 9 mg/L was reduced by 93% compared to controls (Fig 2, Panel B) and the total broodsize EC_50 _for Cd was 3.93 (95%: CI 3.15-4.94) mg L^-1^. A reduction in growth rate and final body length was apparent in worms exposed to >2.5 mg/L of Cd (Fig. 2, Panel C).

DEBtox fits showed no clear dose associated effect of FA on survival patterns over the exposure period (Fig. 2, Panel D). FA did, however, have a negative affect on egg production (Fig. 2, Panel E), although an EC_50 _for broodsize could not be calculated because the maximum reduction found was only 42% at 4 mg/L (n.b. 37% and 39% at 1 and 2 mg/L respectively). FA had an effect on growth patterns over time, with model fits describing slower growth at high FA concentrations, although ultimately worms at all concentrations reached the same body size. (Fig. 2, Panel F).

DEBtox fits indicated that AZ affected survival over time, with steepest mortality curves found at the highest exposure concentrations (Fig. 2, Panel G). DEBtox also highlighted that AZ affected temporal patterns of egg production (Fig. 2, Panel H). Greatest reduction of 56% was at 300 mg/L and the EC_50 _for broodsize was 224 (175 - 273) mg L^-1^. Temporal patterns of weight change indicated slow growth and at higher concentrations and a reduction in final body size (Fig. 2, Panel I).

All growth, reproduction and lifespan data for all exposure concentrations and for all measured time points for DEBtox parameterisation for each compound were used (Fig. 2). Parameter estimates and the best-fitting physiological mode of action are given in Table 1. Log-likelihood ratio values for each of the fitted models are shown in Additional file 1. These values indicate the fit of the different tested models to the data, with the model showing the lowest log-liklihood ratio value having the best fit.

**Table 1:** Parameters derived from DEBtox model fits of time series life-history trait data

Cadmium	Fluoranthene	Atrazine	Physiological
Von Bertalanffy growth rate (d-1)	0.878	1.05	1.27
Initial length (μm)	17.7	24.1	32.4
Length at which ingestion is half of maximum (μm)	30.7	37.4	45.7
Length at start of reproduction (μm)	71.6	86.8	87.0
Maximum length (μm)	132	134	135
Maximum reproduction rate (eggs/d)	130	189	196
**Ageing**			
Maximum total number of eggs	146	152	178
damage killing rate (d-1)	0.00701	0.00504	0.00580
Damage amplification (d-1)	0.196	0.166	0.154
**Toxicological**			
Mode of action	Assimilation	Costs for growth and reproduction	Maintenance
elimination rate (d-1)	0.0811	12.6	1.27
Half-saturation conc. for uptake (mg/L)	27.8	n.e.	63.2
NEC for survival (mg/L)	6.57	n.e.	222
killing rate (L/mg/d)	0.118	n.e.	0.0897
NEC for effects on growth/repro (mg/L)	0.000134	0.000834	13.4
Tolerance concentration (mg/L)	7.53	4320	234
Decrease length at puberty due to chemical stress [-]	0.656	0.240	0.540
Decrease total eggs due to chemical stress [-]	3.68	1.46	14.5

From the DEBtox fits, a set of single parameters for each physiological (e.g. growth rate, maximum reproduction rate), aging related (e.g. damage killing rate, damage amplification) and toxicological (elimination rate, killing rate) parameters were estimated for each of the tested chemicals (Table 1). Of these perhaps the most toxicologically meaningful at the system level is the likely physiological (energetic) mode of action of the compound. This parameter is determined by separate fitting of the DEBtox model for each possible physiological mode of action to identify the effect that gives the best fit of the model to the measured data (see Additional file 1). For Cd, the physiological mode of action that best fitted the data was an effect on energy assimilation. For FA, the best fitting mode of action was costs for growth and reproduction; while for AZ cost for maintenance of somatic tissue gave the best fit (Additional file 1). The DEBtox modelling, thus, suggests that the three tested chemicals which cause some similar life-history changes (e.g. concentration dependent reduction in egg production) and also some distinct changes (e.g. strongly reduced final body size for Cd, reduced growth rate to similar final body size for FA), do so through different physiological mechanisms. The mechanistic aetiology of the physiological changes in resource allocation associated with exposure to each chemical, thus, became the focal point of the transcriptional analysis.

### Transcriptional profiling by whole genome microarray

**Figure 3:**
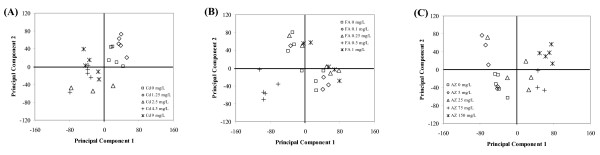
**Principal component analysis**. Scores plot for PC1 and PC2 from a PCA of normalised whole genome microarray data for *C. elegans *exposed to control and 4 concentrations of (A) Cd, (B) FA, (C) AZ.

Successful and repeatable hybridization was indicated by a high correlation (r^2 ^> 0.9) and linear relationship across 2-3 orders of magnitude for the lucidea scorecard samples (see Additional file 2). Initially the effect of exposure to each chemical on the transcriptome was analyzed by PCA of each unfiltered normalized data-set. Distribution and clustering of samples within PCA models indicated that there was no specific impact of exposure on between sample variation for any chemical (Fig. 3). For Cd, the scores plots for PC1 and PC2 of a PCA model with an R^2 ^= 0.4 indicated a separation of the control and 1.25 m g/L samples from the three highest treatments along an axes across the first two PCs (Fig. 3a).

The scores plots for PC1 and PC2 of a PCA model for FA with an R^2 ^= 0.39 indicated a partial separation of samples along the first PC, with the group of samples exposed to 0.5 mg/L identified as outliers (Fig. 3b). Re-analysis of the data-set excluding the 0.5 mg/L samples produced a weaker PCA model (R^2 ^= 0.31), this model did, however, support the presence of a concentration related separation of transcript profiles (see Additional file 3). Because, however, all samples exposed to 0.5 mg/L showed a high similarity and there was no evidence through normalization of a systematic bias in the preparation or hybridization of these arrays, there is no clear and justified reason to exclude these transcript profiles from further analysis. The PC1 and PC2 score plots of a PCA model for AZ with an R^2 ^= 0.31 showed that the control, 5 and 25 mg/L exposed worms were separated from the 75 and 150 mg/L atrazine exposed worms along PC1 (Fig. 3c).

To identify gene expression changes that were associated with the toxicosis of the three chemicals, detailed analysis was undertaken to identify pathways linked to chemical effects at the test concentration that approximated most closely to the EC_50 _for total broodsize for each chemical (2.25 mg/L Cd, 1 mg/L for FA, 150 mg/L for AZ). The predominant approach used was an analysis based on annotation enrichment within a set of differentially regulated genes. This was designed to allow analysis of the changes in expression that were associated with the putative physiological mechanisms of action identified by DEBtox modelling.

**Figure 4:**
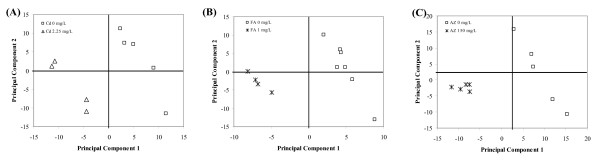
**Partial least squares discriminant of percentage changing genes per GO term at concentrations close to reproduction EC_50_**. Scores plot for PC1 and PC2 from a PLS-DA analysis conducted using the percentage of genes associated with each GO term changing in expression by 1.5 fold at a concentration approximating to the brood size EC_50 _when compared to controls for (A) Cd, (B) FA, and (C) AZ. Only terms represented by 10 or more genes were used for the analysis.

For Cd, a PLS-DA analysis of normalised individual gene expression levels for controls and 2.25 mg/L Cd exposed worms indicated a distinct separation of the samples along PC1 of a highly robust model (Q^2 ^= 0.753) (see Additional file 4a). PLS-DA analysis of the relative percentage of changing genes by biological process GO term indicated a separation of control and four successfully hybridised 2.25 mg/L exposed samples within a highly robust model (Q^2 ^= 0.99) (Fig. 4a). GO terms highly correlated with PC1 included terms associated with cellular organisation (GO:0016043), DNA metabolism (GO:0006259) and also further processes downstream of these effects associated with cell cycle (e.g. GO:0007049) and reproduction (GO:0000003). An indication of effect on energy metabolism was evident by changes in the expression of genes associated with lipid metabolism (GO:0006629)(Table 2).

**Table 2:** GO term classifications correlated with PC1 of a PLS-DA analysis. assessed by GO term classification counts of the 20 Gene Ontology Biological process terms most strongly correlated with PC 1 within a PLS-DA analysis that separates control worms from worms exposed to an approximate EC50 for each of the three tested chemicals.

GO Class ID	Definitions	Cd	FA	AZ
GO:0008150	biological process	20	20	20
GO:0008152	metabolism	5	8	15
GO:0016043	cell organization and biogenesis	4	5	6
GO:0009056	catabolism	1	7	3
GO:0006810	transport	4	4	2
GO:0000003	reproduction	2	2	
GO:0006139	nucleic acid metabolism	3		8
GO:0006996	organelle organization and biogenesis	4		6
GO:0006259	DNA metabolism	1		5
GO:0015031	protein transport		3	1
GO:0007010	cytoskeleton organization and biogenesis	3		
GO:0006629	lipid metabolism	1		
GO:0007049	cell cycle	5		
GO:0005975	carbohydrate metabolism		2	
GO:0009058	biosynthesis			2
GO:0019538	protein metabolism			2
GO:0006519	amino acid and derivative metabolism			6
GO:0006412	protein biosynthesis			2

A second approach to annotation enrichment analysis used the Database for Annotation, Visualization and Integrated Discovery (DAVID) tool. This web based resource provides a set of functional annotation tools for identification of biological meaning within differentially regulated gene lists. DAVID input for Cd was a list of 2326 genes identified as significantly up or down regulated between the 0 and 2.25 mg/L Cd exposure without multiple sample correction (against an expectation of 937 by chance) (n.b. with false discovery correction [20] applied, only one gene (predicted gene ID Y59A8B.12) of unknown function was significant). The list without multiple sample correction was used as DAVID input to limit type II errors in significant gene identification. The fact that the DAVID pathway enrichment analysis system itself includes an algorithm for multiple sample correction effectively limits type 1 errors during identification of enriched pathways.

The DAVID analysis for Cd identified three KEGG pathways that were significantly over represented in the 0 vs 2.25 mg/L gene-list. These related to glutathione metabolism (5 of 12 present genes), taurine and hypotaurine metabolism (2 of 2 present genes) and protein export (4 of 10 present genes). DAVID analysis also identified a number of significantly enriched terms contained within the annotation clusters (Table 3). A number of the most highly enriched of these clusters, such as membrane association, cation binding, glycoprotein signalling, transcription and protein kinase activity were also significantly enriched in both the FA and AZ exposed worms. Cd also shared two annotation clusters containing significant terms with FA. These related to EGF domains and isomerase activity. Four annotation clusters were shared between Cd and AZ exposed worms, of which protein chaperoning and mitochondria associated genes were the most strongly enriched. Three of the significant annotation clusters were unique to Cd exposed worms. These related to transcription factor activity, transmembrane transport and thiol proteases.

**Table 3:** DAVID Functional Annotation Clustering output for genes significantly differently expressed compared to controls following exposure to an approximate EC50 for each tested chemicals.

	Cd	FA	AZ
***Cd_FA_AZ***			
membrane/transmembrane	8.61	7.85	1.39
cation binding	5.08	2.35	4.22
glycoprotein signalling	4.06	3.25	2.93
transcriptional activity	2.88	0.93	1.75
protease activity	2.64	1.04	1.6
protein kinase activity	1.58	1.04	2
signal tranduction activity	1.15	1.51	1
ribosomal protein	0.87	3.75	4.12
***Cd_FA***			
isomerase activity	1.55	1.35	
EGF-like domain	0.94	0.95	
***Cd_AZ***			
mitochondria	1.59		1.57
homeobox	1.48		1.23
collagen	0.67		0.63
stress and heat shock chaperoning	2.28		1.49
***FA_AZ***			
wd repeat		2.35	3.72
nucleosome core and chromatin		1.36	2.13
haem/monooxygenase		2.3	1.47
ion transport		0.57	0.58
***Cd***			
transcription factor activity	2.28		
tansmembrane transport	2.21		
thiol protease	0.75		
***FA***			
oxidoreductase		3.96	
mRNA splicing		2.83	
amino acid biosynthesis		1.21	
flavoprotein		0.95	
protein biosynthesis initiation		0.63	
protein biosynthesis tRNA synthesis		0.6	
***AZ***			
larval development			5.1
magnesium ion binding			1.5
glycolosis			1.45
threonine protease			0.97
calcium			0.83
acyltransferase			0.82
ubiquitination			0.76

**Figure 5:**
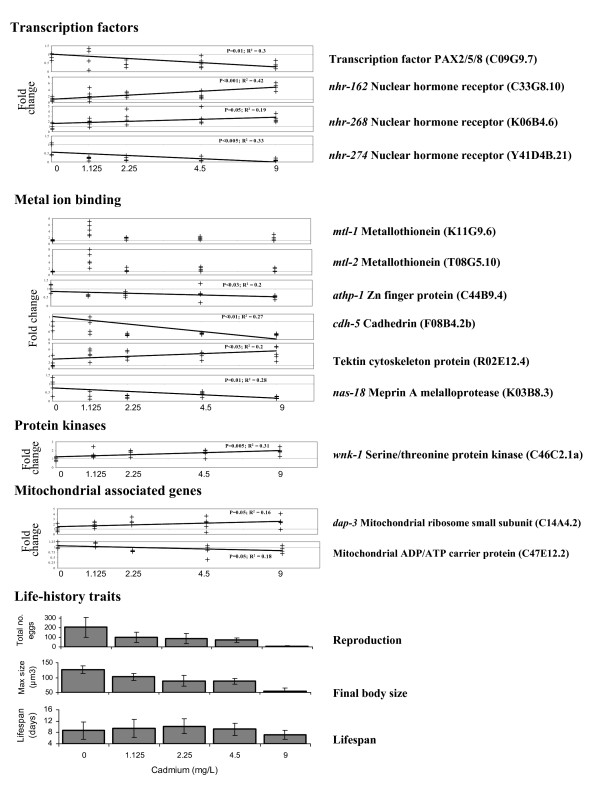
**Life-history and expression of key genes in Cd exposed nematodes**. Transcriptional responses for a series of selected genes associated with ontology clusters significantly enriched for differentially expressed genes between adult *C. elegans *exposed to a control and 2.25 mg Cd/L agar. Array data were normalised per chip and per gene median polishing and are expressed relative to the control samples. Error bars indicate the SD of the mean.

Expression patterns of individual genes that were associated with the significant annotation terms were investigated across the full exposure range, This was done to identify a delineative sub-set of annotated changing genes that showed a significant linear relationship with Cd exposure concentration as opposed to only possibly idiosyncratic expression change at the approximate EC_50 _concentration for which the gene list for pathway analysis was generated (Fig. 5). Such gene level scrutiny, therefore, provided insight into the mechanistic effects of Cd exposure that allowed a deeper assessment of the transcriptional changes associated with Cd exposure.

One category of genes within which a number of transcripts showed a significant linear relationship of expression with Cd exposure was the transcription factors. Genes within this category showed both significant up and down regulation. These included Zn finger type factors such as *athp-*1 and also nuclear hormone receptors such as *nhr-162*, *nhr-268 *and *nhr-274 *(Fig 5). The effect of Cd exposure on these multiple transcriptional factors was indicative of a wider remodelling of the transcriptome as shown by the changes in expression profiles across treatments illustrated within the PCA (Fig. 3a). Among the metal binding genes responding to Cd were *mtl-1 *and *mtl-*2, both of which code putative metallothioneins. These two genes were upregulated in all Cd treatments (Fig. 5). Expression of a number of structural proteins was also related to Cd exposure. These include the cadhedrin *cdh-5 *which was down-regulated and the tektin cytoskeleton protein ecoded by sequence R02E12.4 which was strongly upregulated. Expression of multiple meprin metalloproteases (e.g. nas-18) and E3 ubiquitin ligases that are involved in protein tagging and degradation were also significantly affected by Cd (Fig 5). Another category of genes highlighted as being responsive to Cd exposure were protein kinases. Here of nine significant genes, eight such *wnk-1 *were upregulated. Expression of genes with specific involvement in mitochondrial biogenesis and functioning, such as *dap-3 *and ADP/ATP carrier protein was also significantly related to Cd concentration indicating an effect of exposure on mitochondrial integrity.

For FA exposed worms, PLS-DA analysis of individual normalised gene expression data of the controls and 1 mg/L exposed samples indicated a clear separation along PC1 of a highly robust model (Q^2 ^= 0.993) (Additional file 4b). The PLS-DA analysis of relative percentage of changing genes per GO biological process term, also showed a clear separation of controls from the 1 mg/L exposed samples (Q^2 ^= 0.99) (Fig. 4b). GO biological processes represented among the terms most negatively correlated with PC1 for FA exposed worms included ontologies associated with catabolism (GO:0009056) and cell organisation and biogenesis (GO:0016043): terms which were also identified by the same analysis for Cd and AZ (Table 2). Terms that were particular associated with FA exposure included protein trafficking (GO: 0015031) and carbohydrate metabolism (GO:0005975).

Functional annotation analysis using DAVID was conducted with a list of 1807 genes significantly differentially expressed (p < 0.05) without multiple sample correction between the control and 1 mg/L FA treatments. False discovery correction according to Benjamini and Hochberg [20] produced a list of 38 significant transcripts. Based on the uncorrected list, DAVID identified six KEGG pathways that were significantly over represented in differentially expressed genes. These related to glutathione metabolism (5 of 12 genes present), metabolism of xenobiotics by P450 (16 of 22 genes present), lycine, serine and threonine metabolism (6 of 20 genes present), nitrogen metabolism (4 of 10 genes present), alkaloid biosynthesis (2 of 2 genes present) and the ribosome (20 of 74 genes present).

DAVID analysis also identified a number of annotation clusters that contained a significant overrepresentation of genes differentially expressed following FA exposure (Table 3). In addition to clusters shared with both Cd and AZ exposed worms and also with Cd exposed worms as outlined above, annotation terms were identified that were shared only by FA and AZ exposed worms. These included annotations associated with DNA structure and also haem/monooxygenases: which suggest a role of cytochrome P450s in the metabolism of these two organic chemicals. Six annotation clusters with significant terms were also identified that were unique to the FA exposure. Among these oxidoreductases were most enriched, while clusters associated with both mRNA transcript processing and the biosynthesis of amino acid and proteins that are symptomatic of an effect of FA on macromolecule turnover were also significantly enriched.

**Figure 6:**
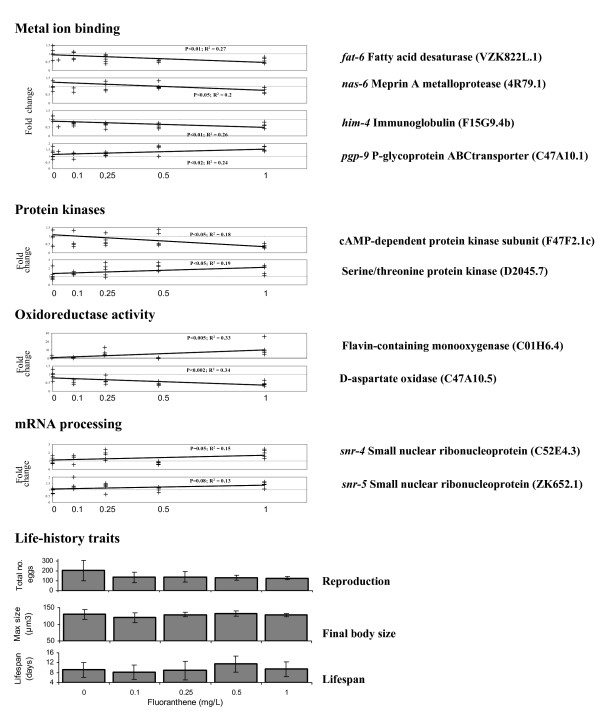
**Life-history and expression of key genes in FA exposed nematodes**. Transcriptional responses for a series of selected genes associated with ontology clusters significantly enriched for differentially expressed genes between adult *C. elegans *exposed to a control and 1 mg FA/L agar. Array data were normalised per chip and per gene median polishing and are expressed relative to the control samples. Error bars indicate the SD of the mean.

For annotation terms identified as significantly enriched for genes differentially regulated by FA, patterns of expression of individual genes associated with each term were investigated across the exposure range (Fig. 6). A number of the genes showing dose related expression change were involved in metal ion binding (a common term with Cd and AZ exposed worms). Genes in this category responsive to FA included transcripts associated with macromolecule turnover, such as the fatty acid desaturase *fat-6 *and the meperin metalloprotease *nas-6*, immunoglobulins such as *him-4 *and substrate transport, such as the P-glycoprotein ABC-transporter pgp-9 (Fig 6). This latter gene is known to be involved in xenobiotic transport within the cell. A further category of genes responsive to FA, as well as Cd and AZ, exposure were the protein kinases. Three genes in the category were down regulated,(e.g. cAMP-dependent protein kinase) and two upregulated (e.g. Serine/threonine protein kinase) at 1 mg/L FA. Genes involved in oxidoreductase activity differentially expressed following FA exposure included transcripts coding flavin containing monoxygenase that are involved in oxidative stress protection, as well as downregulated genes involved in dehydrogenation such as D-aspartate oxidase. Genes involved in mRNA processing that were differentially expressed included multiple small nuclear ribonucleoprotein (e.g. snr-4 and snr-5), all of which were upregulated at 1 mg/L FA (Fig. 6).

For AZ exposed worms, PLS-DA analysis of normalised gene expression data for the controls and 150 mg/L AZ exposed worm indicated a separation along PC1 of a highly robust model (Q^2 ^= 0.998) (Additional file 4c). PLS-DA analysis of the relative percentage of changing genes per biological process GO term also indicated a clear separation of control and 150 mg/L exposed samples along PC1 (Q^2 ^= 0.985) (Fig. 4c). Analysis of PC1 loading to identify the most negatively correlated terms indicated a strong representation of terms associated with nucleic acid metabolism (GO:0006139) and organelle organisation and biogenesis (GO:0006996), a response that was shared with Cd exposed worms (Table 2). Unique terms that were associated with AZ exposure included protein biosynthesis (GO:0006412) and metabolism (GO:0019538), as well the metabolism of individual amino acids (GO:0006519). These responses are all indicative of a potential effect of AZ on protein turnover.

Functional annotation analysis using DAVID was conducted using a list of 1333 genes significantly differently expressed (p < 0.05, without false discovery correction) between the 0 and 150 mg/L AZ treatments. Multiple sample correction according to Benjamini and Hochberg [20] reduced this list to 47 significant genes. From the longer gene list, DAVID analysis identified three KEGG pathways that were significantly over represented. These were glycolysis (7 of 19 present genes), pyruvate metabolism (8 of 14 present genes) and the ribosome (18 of 74 present genes).

A number of significantly enriched annotation clusters were also identified by DAVID analysis (Table 3). Clusters shared with both Cd and FA, for which AZ worms also showed a high level of enrichment of differentially regulated genes were for cation binding, protein synthesis and intracellular signalling. Enriched terms shared with Cd exposed worms were associated with mitochondrial function, body patterning and stress and heat shock, while those shared with FA were associated with oxidoreductase activity and DNA integrity. Seven enriched annotation clusters were uniquely associated with AZ exposure. Notable among these was larval development which was the most strongly enriched cluster among the significant terms. Glycolysis, and also terms associated with protein tagging and degradation were also significant.

**Figure 7:**
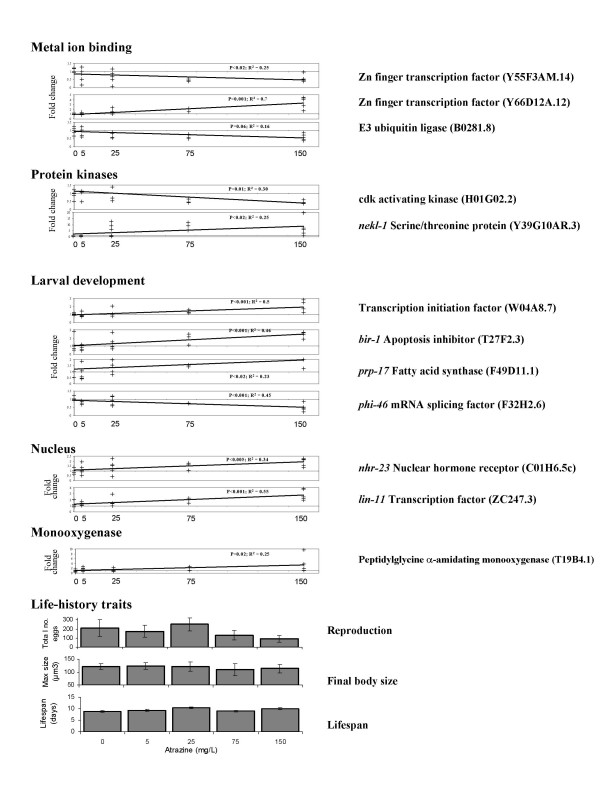
**Life-history and expression of key genes in AZ exposed nematodes**. Transcriptional responses for a series of selected genes associated with ontology clusters significantly enriched for differentially expressed genes between adult *C. elegans *exposed to a control and 150 mg AZ/L agar. Array data were normalised per chip and per gene median polishing and are expressed relative to the control samples. Error bars indicate the SD of the mean.

Specific genes that were associated with annotation terms highlighted for AZ that showed a significant linear relationship of expression with exposure concentration included genes associated with metal ion binding such as numerous zinc finger transcription factors, as well as transcripts encoding proteins involved in protein tagging and degradation such as Meperin metalloproteases and ubiquitin ligases (Fig. 7). Protein kinases responding to AZ exposure, included both down regulated genes, such as the cdk activating kinase encoded by sequence H01G02.2 and upregulated genes such as the serine/threonine protein kinase *nekl-1*. A large number of changing genes were associated with the larval development term. These included histones, transcription factors such as *taf-1 *which encodes a factor that possesses histone acetyl transferase (HAT) activity as well as genes involved in mRNA splicing, the control of apoptosis and macromolecule (e.g. lipid) metabolism (Fig. 7). A number of changing genes were also associated with nuclear activity, including numerous transcription factors, such as *lin-11 *and nuclear hormone receptors, such as *nhr-23*. Differentially expressed monooxygenases included peptidylglycine alpha-amidating monooxygenase (Fig 7), which showed a significant increase with exposure, indicating an effect of AZ on the nematode p450 system.

## Discussion

The genetic control of life-history and the responses of traits to stress is a fundamental aspect of biology that is of physiological and ecological significance [2,21]. The approach taken by molecular geneticists to understand life-history events like growth, reproductive timing, reproductive output and life-span has mostly been through reverse genetics. In particular, thanks to deletion strains and the use of knock down methods, it has been possible to identify a range of genes and small metabolite determinants that effect the life-history of laboratory model species such as *Saccharomyces cerevisiae*, *Drosophila melanogaster*, *Arabidopsis thaliana *and *C. elegans*, [3,22-24]. As an example, loss of function mutants for genes in the insulin signaling pathway have been shown to extend lifespan in *C. elegans *[25,26] and also confer reduced sensitivity to environmental stress [27-30]. This suggests the presence of a functional link between stress responses, such as the processing and detoxification of toxic chemicals, and the timing of life-cycle events including death [31].

For ecologists, the notion of an association between (chemical) stress exposure and the control and timing of life-history events is consistent with existing and well developed concepts. From an ecological perspective, life-history can be understood through the use of physiologically based models that describe how organisms acquire resources from food and subsequently allocate these to key biological processes, such as growth, maintenance, and reproduction [14]. Further, by linking rules for metabolic organization within dynamic energy budget theory with pharmacokinetic models that describe chemical uptake and elimination, it is possible to link the hazards associated with chemical exposure to resulting toxic effects [32]. This link is described within the DEBtox framework which provides a physiologically based context for understanding the mechanistic and energetic basis of toxicant effects.

Here, data from three continuously monitored life-cycle toxicity tests with *C. elegans *were used to parameterize DEBtox models from which a number of biologically meaningful parameters describing toxicity, including toxicokinetic and life-history associated parameters and physiological mode of action, were estimated. The life-cycle toxicity tests themselves and subsequent DEB modeling indicated that the three tested chemicals each had effects on a range of life-history traits. Further, even though time dependent patterns of survival, growth and reproduction were affected by each chemical, the DEBtox analysis indicated that the underlying physiological mechanisms causing these effects were different in each case.

To provide initial insight into the energetic basis of life-history effects, DEBtox model fits were used to allow calculation of a number of physiological, ageing related and toxicological parameters (Table 1). Of these perhaps the most toxicologically meaningful is the physiological (energetic) mode of action of the compound which is determined by serial fitting of separate models. For Cd the physiological mode of action that best fit the data was an effect on energy assimilation. This supports the results of previous DEBtox analysis of Cd effect in *C. elegans *[33,34], as well as the results of similarly designed studies of Cd toxicity for the springtail *Folsomia candida *[18], and *Daphnia magna *[35]. An effect of Cd on energy acquisition in *C. elegans *is also consistent with the observation of reduced feeding following Cd exposure [36,37].

For FA, the best fitting physiological mode of action within DEBtox was costs for growth and reproduction. This mode of action suggests that the production of somatic and reproductive tissue becomes most costly due to FA exposure, meaning that growth (and egg production) is slowed, but that also ultimately the same final body size of adult worms is reached. These model predictions for the an effect of FA exposure on costs for growth and reproduction are consistent with the mode of action observed for pentachlorobenzene in *C. elegans *[37], suggesting this may be a generic physiological mode of action for narcotic organic chemicals in this species.

For AZ, the best fitting physiological mode of action within DEBtox was costs for somatic and/or maturity maintenance. These results, to our knowledge, represent the first DEBtox based assessment of the physiological mode of action of any triazine herbicide. This mode of action suggests that AZ exposed nematodes are required to divert an increased proportion of available energy to protect or repair somatic and reproductive tissues.

To address the specific mechanistic aetiology of the physiological changes in resource allocation associated with the toxicity of each chemical, transcriptional profiling using a whole genome oligonucleotide microarray was used. This was not the first study that has used microarray analysis to study the effects of chemical exposure on the *C. elegans *transcriptome [38-41]. Uniquely, however, in this study we were able to link observed transcriptional changes with modelled energy budget responses as detailed through DEBtox fits. This combined analysis provides better phenotypic anchoring for the interpretation of the transcriptional changes that are associated with chemical exposure.

Of course while the DEBtox analysis did indicate particular chemical effects, some common responses were seen as reflected by the identification of a number of common pathways and annotation terms that were enriched for genes differentially regulated following exposure to all chemicals. These common enriched annotations included membrane proteins, cation binding, protein kinase activity/signal transduction, ribosomal proteins and protein degradation. Many of these pathways, such as changes in cation trafficking [42], mitogen-activated protein kinase signalling [39,43] and protein chaperoning [40,44], have been previously identified as responsive to chemical exposure in *C. elegans*. Moreover in yeast, studies have identified a group of "common environmental response" genes, the expression of which were ubiquitously altered by a range of environmental stressors. When viewed at the pathway rather than individual gene level [45], many of the common responses found in this study are consistent with pathways such as heat shock, organic compound metabolism, oxidative stress, protein turnover, metal transport, thioredoxin/glutathione regulation and energy generation that were highlighted as ubiquitously stress responsive in yeast [46,47].

In addition to transcriptional responses shared between all chemicals, further differentially expressed gene enriched annotation terms were also identified by DAVID analysis that were common between particular chemical pairs. These included the KEGG pathway for glutathione metabolism, a peptide that is involved in protection of cells from chemical injury resulting from oxidative stress [13] for both Cd and FA; and haem/monoxygenase metabolism for the FA and AZ. The later indicates a likely role of cytochrome P450 in the detoxification of these two organic chemicals. For FA this potential role for P450 was supported by the differential regulation of many (16 of 22) genes in *C. elegans *that are associated with the KEGG pathway for metabolism of xenobiotics by P450. Both the glutathione and monooxygenase pathways have been previously implicated as core to the responses of many species to a range of chemical stressors [13].

As well as common responses, unique transcriptional changes were also seen that when culminating in overrepresentation at the pathway level potentially contribute to the specific aetiology of the life-history effects of each chemical. For Cd, DEBtox indicated that the most plausible mode of action was a reduction in energy assimilation. At the mechanistic level, the most obvious manifestation of this effect would be down-regulation of biological processes involved in sugar and lipid metabolism, electron transport, ATP turnover and mitochondrial function. Although the mitochondria were highlighted as a target of Cd (and also FA), there were other annotation clusters that actually showed a higher degree of enrichment for differentially expressed genes in Cd exposed worms. One such category of genes were associated with cation binding. These included transcription factors and structural proteins, as well as the two putative metallothionein *mtl-1 *and *mtl-2 *which have been functionally linked to Cd responses in *C. elegans *[48,49]. Other gene expression response associated with Cd exposure included membrane associated genes and also transcriptional activity, with nucleic acid metabolism also highlighted. Previous work has implicated Cd in DNA damage in earthworms [50], nematodes [43] and mammalian cell lines [51]. Further, a global down regulation of RNA production has also been identified in *C. elegans *exposed to Cd [52]. These findings, coupled with the indications from our transcriptional analyses, raise the possibility that Cd may affect DNA integrity and transcription, thereby causing a reduction in transcription and protein synthesis that ultimately leads to a suppression of energy demand. Such effects would be manifested as an overall change in energy assimilation, but with the effect coming from suppressed demand rather than reduced energy production.

For FA, the best fitting DEBtox physiological mode of action was costs for growth and reproduction. This suggests that worms exposed to FA may have to expend a greater amount of their available energy to the production of new biomass. The ontology term and pathways analysis for FA exposed worms provided convincing support for an effect of FA on such production costs. Thus DAVID analysis indicated a specific effect of FA on the synthesis of amino acids and proteins and a strong effect on the ribosome, the site of protein production; while the PLS-DA analysis highlighted effects on catabolism, including genes associated with the KEGG pathway for protein transport. These effects all suggest a possible effect of FA on protein synthesis and turnover.

A previous metabolomic study of the effects of pyrene (another PAH) on earthworms indicated increased protein breakdown as a result of decreased glucose and fatty acid metabolism [53] and similar responses have been seen in rats during toxicological studies [54]. This suggests that energy metabolism may be switched from carbohydrates to protein possibly as a result of an increase in the amount of free amino acid present in cells as a result on an increase in the degradation of protein compromised by chemical exposure. This will result in the need for frequent re-synthesis of key structural proteins during growth, development and egg production at a considerable energetic cost to the organism.

For AZ, the best fitting physiological mode of action within DEBtox was costs for maintenance of somatic and reproductive tissue. Without further insight it may be hypothesized that these costs are likely to be linked with an increase in the protection of the range of *C. elegans *proteins involved in detoxification, antioxidant protection and protein chaperoning [55]. While AZ worms did show significantly enriched differential expression of monooxygenases, including a number of genes involved in antioxidant defense, it was noticeable that many of the primary components of the antioxidant protection systems, such as SOD, catalase, glutatione peroxidase, peroxiredoxins, were not differentially expressed as a result of AZ exposure: at least at the transcriptional level at the sampled time-point.

Based on the annotation analysis of genes differentially expressed by AZ exposure, the most strongly enriched categories were transcripts associated with larval development. Within this category, many of the differentially expressed genes were linked with DNA integrity, such as the expression of histones, and genes involved in transcriptional regulation and mDNA splicing. Further within both the PLS-DA and DAVID analysis, an enrichment of differentially expressed genes associated with DNA integrity and repair was indicated. This is all consistent with previous work on atrazine which has shown a link between atrazine exposure and DNA damage [50,56,57]. This suggests that increased demands to maintain somatic and reproductive tissue may be associated not only with demands for organic compound metabolism, but also with the repair of DNA damage. Such repair, which is necessary to maintain DNA integrity and reduce rates of cellular apoptosis, requires the use of energy resources that ultimately need to be diverted from other processes.

To fully understand the effects of chemical stress on life-history responses, such as growth, reproduction and longevity, there is a need to link the effects of chemicals at the detailed mechanistic level with higher organisation changes in resource allocation and trait performance. Process based models such as DEBtox can provide a framework to understand the physiological basis of life-history in terms of energy allocation. Physiological mode of action predictions from DEBtox can provide a useful indication of the basis of the toxic effect. However, these modes of action still represent changes in very broad processes. Transcriptional profiling on the other hand can provide detailed understanding of the target site and resulting effects of a chemical, but not how these expression changes combine to cause effects on the biological response of major life-cycle traits for the whole organism. Combined, though, as has been done here, the two approaches can provide a unique view of both the range of gene expression response associated with chemical exposure and the culminating effects on resource allocation and life-history.

The work that is presented here illustrates a stepped approach that utilises both physiological based modelling and gene expression profiling to probe the energetic basis and aetiology of chemical effects on demographic traits. The analysis as conducted essentially represents a two stage process in which DEBtox analysis is first used to generate hypotheses concerning the physiological mode of action of the tested chemicals. Gene expression profiling is then used to investigate the validity of these hypotheses in terms of biological processes and individual genes.

Clearly the approach to data modelling and interpretation used represents only one of a number of possible ways that the comprehensive demographic and transcriptional data-sets can be analysed. One potential alternative approach could have been to include all expression data and life-history measurements within a single analysis to look for correlation between trait measurements and gene expression change. While this method could have been used to look for associations between gene expression and individual traits, such an approach is not yet possible within a DEBtox framework since all trait data is included within the model to generate only a single set of DEBtox parameters per chemical. To undertake an analysis that allows correlations between DEBtox parameters and gene expression, similar data-sets for further chemicals would be required to allow robust correlations to be made. To allow the wider toxicogenomic community the opportunity to apply different approach to the analysis of the results described here, all data has been made accessible in a MIAME compliant format using the advanced path of data dissemination, namely via MIAME/Env (for details see: nebc.nox.ac.uk/miame/miame_env.html). Storage in the MIAME/Env format allows unrestricted (open access) in support of further deep mining of the data.

## Conclusions

Linking molecular mechanisms of toxicant to their physiological mode of action is not just of academic interest. Within applied toxicology understanding the cascade through which chemicals exert their effects can be important for toxicant categorisation, development of structure activity relationships, and identification of the most appropriate models for describing the joint effects of chemical mixtures [58]. Here we have demonstrated the value of adopting a combination of top down process based modelling and a bottom up mechanistic approaches to identify physiological modes of action of toxicants and associated gene expression changes. Through process based modelling it was identified that three xenobiotics from different chemical classes, produce some similar effects, but also caused specific life-history responses that could be linked through process modelling to effects on resource allocation. Transcriptional profiling allows the further identification of these effects in terms of induced gene expression change and their relationship with exposure concentration. Such detailed assessment of life-history effects and their causes can provide a platform for a more detailed categorisation of chemicals for appropriate risk assessment and a sound mechanistic basis for the identification of effect linked markers of chemical injury.

## Methods

### Experimental design

Three separated experiments were conducted, one with each chemical, with each experiment designed to measure both the effects of exposure on a range of life-history traits and also to obtain a sample of exposed nematode for transcriptional profiling (see Fig. 1 for schematic of experimental design). All experiments were conducted with C. elegans strain cib-1(e2300)I., a temperature sensitive mutant that is wild-type below 25°C, but displays an embryonic lethal phenotype at 25°C and above.

For the exposure worms were reared during pre-exposure phases at 15°C, a temperature with no effect on embryonic lethality. The main exposure was conducted using eggs synchronised by bleaching from the pre-exposed worms with the temperature switched to 25°C, at temperature at which embryonic lethality is induced within the first few cell divisions. when the resultant offspring reached L1 stage. This design meant that a cohort of worms could be reared throughout the main exposure for their entire life-span, thereby provide suitable data for DEBtox modelling, without the population used for transcriptional profiling becoming infiltrated by hatched and growing juveniles as would have been the case if a wild-type strain was used. The advantage of this was that the transcriptional profile gained from the analysis of exposed replicated cohorts of worms reared to adult could be linked to measurements of life history traits (growth, maturation, reproduction and mortality) recorded from similarly kept individuals without any influence of the presence of different worm stages in the sample on measured expression pattern. Thus, all samples generated differed only in relation to chemical exposure rather than stage specific effects within the population.

### Life-cycle toxicity tests

Dosing of the NGM agar and OP50 were conducted using analytical grade cadmium chloride (Cd) (Sigma Chemicals, Poole, UK), fluoranthene (FA) (Fisher Chemicals, Loughborough, UK) and atrazine (AZ) (Sigma, UK). Stock solutions were prepared in bi-distilled water, ethanol and dimethyl sulfoxide (DMSO) respectively. Stock solutions were added to NGM cooled to 55°C, to give a total of 12 test concentrations per experiment. Concentrations were selected based on prior information about toxicity and chemical solubility derived from range finder tests. The test concentrations used were: Cd 0, 0.141, 0.281, 0.563, 1.125, 1.688, 2.25, 3.375, 4.5, 5.625, 6.75, 7.875, 9 mg/L; FA - 0, 0.025, 0.05, 0.075, 0.1, 0.175, 0.25, 0.375, 0.5, 0.75, 1, 2, 4 mg/L; AZ - 0, 1, 2.5, 5, 10, 25, 50, 75, 100, 150, 200, 250, 300 mg/L. For the FA and AZ tests, equal amounts of solvent (0.04% ethanol or 0.25% DMSO) were added to all treatments including controls.

For each experiment, initially a stage synchronised population was obtained by bleaching culture plates of gravid adults. Aliquots of extracted eggs were then transferred to multiple NGM plates dosed to each test concentrations and previously inoculated with OP50. These eggs were allowed to hatch and the worms grown to adult at 15°C. Each exposed population was then synchronised again by bleaching. These resultant eggs (derived from life-time exposed parents) were then transferred to each of five replicate freshly dosed 90 mm diameter NGM plates for each of the 12 test concentrations per experiment (seven for FA controls). This time the eggs were hatched and at L1 stage switched to 25°C and grown to L2/L3 stage. For each test concentration, 48 individual worms were then taken at randomly from the five replicate plates per treatment and each transferred to a separate well of a 12-well plate dosed to the same test concentration. These plates were then kept also at 25°C and subsequently worms transferred daily to a fresh similarly dosed well. At each transfer, worm survival was checked and the number of eggs counted. Every 6 hours throughout the exposure, the growth of 10 random individuals per treatment was recorded by photographing worms using a Nikon Coolpix 4500 digital camera. Size was measured using Image Pro Express software (Media Cybernetics, Marlow, UK) and volumetric length (cubic root of body volume) calculated [34].

### Microarray analysis

In parallel with monitoring of the single worms, the remaining approximately 10,000 worms that were on the five initial replicate large plates per treatment were kept also at 25°C and periodically checked to ensure they developed simultaneously with the individual worms at the same concentration. At 12 hours after onset of egg production, to ensure stage synchronisation across all samples, all replicate plates from five concentrations that spanned the exposure range used in the life-history study (Cd: 0, 1.125, 2.25, 4.5, 9 mg/L; FA: 0, 0.1, 0.25, 0.5, 1 mg/L; AZ 0, 5, 25, 75, 150 mg/L), were washed in M9 and worms collected by gravitational isolation. This provided a sample of exposed worms for each replicate. The worm samples were immediately frozen in liquid nitrogen and stored at -80°C.

Global RNA transcript abundance was analysed using oligo microarrays printed using *C. elegans *oligonucleotide set version 1.1 (Operon™). Probes were designed from the WormPep DNA Release 75 database, maintained and developed by the Sanger Institute (Cambridge, UK). This set comprises 19,873 70 mer oligonucleotides designed to the 3' region of all predicted genes. The oligonucleotides were printed onto UltraGAPS™ (Corning, Barry, UK) slides at a concentration of 15 μM. Appropriate landmarks of 8 replicates of the Lucida Scorecard (Amersham) were across slides sub-array. The Lucida Scorecard is a selection of heterologous gene reporters which show no cross reactivity to *C. elegans *transcripts. Analysis of calibrators exploited 10 "alien" RNA spikes (components of the Amersham Lucidea Scorecard) introduced at known concentrations, between 1 pmol and 30 nmol, prior to labelling and each hybridized to replicate reporter spots on the array. Image analysis of the signals generated by these reporters was performed for each array to determine the sensitivity and relationship between RNA concentration and fluorescent signal. All oligonucleotides were immobilised by baking at 80°C for 2 hours and UV cross linked. Slides were blocked using a blocking solution of 1% Bovine Serum Albumin, 5 × SSC and 0.1% SDS (all Sigma, UK) at 42°C for 30 minutes, washed five times in sterile water and dried.

Sample RNA was isolated (Tri-reagent, Sigma, UK), cleaned (RNeasy Mini kit, Qiagen, Crawley, UK) and quantified spectroscopically. All experiments were conducted following a reference design with the reference sample compiled from a mixture of RNA extracted from control and Cd, FA and AZ exposed worms. Use of this reference was intended to provide optimal coverage of the spotted genes. For hybridisation, sample and reference mRNA was purified from 15 μg total RNA using Qiagen columns. RNAs were indirectly labelled with fluorescent dyes, reference Cy3 and samples Cy5 respectively and reverse transcribed using Moloney Murine Leukaemia Virus reverse transcriptase (ABgene, Surrey, UK). Cy3 and Cy5 labelled cDNA was purified using the CyScribe™ GFX Purification Kit (Amersham Biosciences, Buckinghamshire, UK). cDNA labelling quality was assessed by scanning 1 μl of sample run on a slide gel under a green (560 BP filter) and red (675 BP filter) laser on a GENETAC™ LS IV scanner (Genomic Solutions, Huntingdon, UK). Hybridisations used 20 pmol of Cy dye molecules for each channel at 42°C for 24 hours. After removal and washing, slides were scanned at 633 nm (Cy5) and 543 nm (Cy3) on a ScanArray™ Express HT microarray scanner (Perkin Elmer, Beaconsfield, UK).

### Data modelling and statistical analyses

A modified version of DEBtox [32], a model based on DEB theory [14] was used to analyse effects on life history traits and to discern the physiological mode of action of each toxicant, as well as a range of other physiologically relevant parameters associated with the toxicokinetic and toxicodynamics of the chemical. Within the DEBtox framework five models have been defined, which represent different energetic modes of action through which chemicals may exert their toxic effect. These are assimilation (less energy acquired from food); maintenance (increased energy demand to maintain existing somatic tissue); costs for growth and reproduction (increased energy demand for production of new somatic tissue and reproductive structures); costs for reproduction (increased energy demand for production of progeny); and hazard to embryo (direct effect on reproduction such as a direct toxic effect on embryo survival).

To fit these variants, the basic DEBtox model described by Kooijman and Bedaux [17] was extended to deal with life-cycle experiments; thereby allowing simultaneous modelling of multiple traits (Jager et al., 2004). Modifications were needed to deal with the specific details of the *C. elegans *life cycle [19,33]. All DEBtox models were fitted using maximum likelihood estimation in Matlab^® ^version 7.0 (Release 14). In all fits, reproduction data only from the period t < 6.5 days were included, as egg production typically stops after this time even in exposed worms and so little information is gained by inclusion of data from further time points. For fluoranthene all models were fitted without including data from the highest exposure concentration (4 mg/L) due to concerns that this concentration exceeded the water solubility limit for the chemical within the test system (crystals observed in system). Results from the fits of the five single DEBtox models were compared, with the lowest log-likelihood values taken to represent the most plausible physiological mode of action for each chemical.

The whole genome microarray data was imported into GeneSpring 7.3 (Agilent Technologies, Stockport, UK). Background measurements were removed and metadata associated with effects on individual worms at each exposure concentration assigned to each array. Normalisation was then performed using Edwards background subtraction and a tip lowess/scaled normalisation. Values were then divided against mean control expression. Quantity assessment was performed by visualising a box plot of the normalised data. This allowed identification of abnormally distributed samples that may be representative of compromised samples or hybridisation. From the exposure concentrations selected for detailed analysis, only one such abnormally distributed sample of 2.25 mg/L Cd was identified. This single sample was not included in any subsequent data analyses.

Initial analyses of the normalised data were conducted using pattern recognition analyses SIMCA-P, version 10 (Umetrics, Umeå, Sweden). Principal component analysis (PCA) was used to identify biologically meaningful patterns of transcriptome remodelling. All PCA models generated were cross validated using an iterative procedure in which the model is rebuilt using only 6/7 of the data as a training set and this is then used to predict the class of the remaining 1/7. From this, R^2^X (the fraction of the sum of squares of the X-matrix explained by the model) can be calculated. Typically, a robust PCA model has an R^2^X > 0.5.

Identification of the major pathways involved in toxicant response was restricted to a comparative analysis at an exposure concentration as close as possible to the EC_50 _for total brood size for each chemical. Comparison at close to the brood size EC_50_, rather than at higher or lower effect levels was selected since at this exposure level, substantive chemical induced remodeling of the transcriptome of consequence for life-history traits can be expected without apoptotic and necrotic process dominating the signal. Samples selected for the analysis were all successfully hybridized replicates from the Cd 2.25 mg/L (57% reduction in brood size), FA 1 mg/L (37% reduction in brood size) and AZ 150 mg/L (56% reduction in brood size) treatments, as well as all control samples for each test chemical.

To identify the pathways involved in biological response at approximate EC_50 _levels, two analytical approaches were used, each utilisng gene ontology as a tool for interpretation of expression change. Firstly, the percentage of genes associated with each GO term changing in expression by 1.5 fold or more in comparison to mean control expression were initially identified for each slide. Genes in this list were then assigned to relevant GO terms and for those terms represented by 10 or more genes, the percentage of 1.5 fold changing genes per term was then compared to the average percentage change. Values of relative change per GO term against mean were then subject to partial least squared discriminant analyses (PLS-DA) using SIMCA-P. All PLS-DA models were cross validated allowing the Q^2 ^(the fraction of the variation in both the X- and Y-matrices explained by the model), as well as R^2^X and R^2^Y (the fraction of the sum of squares of the Y-matrix explained by the model) values to be calculated (a robust PLS-DA model typically has a Q^2 ^score > 0.4, while a Q^2 ^> 0.7 indicates a highly robust model). After confirming separation of control and exposed samples by scores plot analysis, loading plots were reviewed to identify the 20 GO terms most strongly associated with sample separation along the most relevant PC. These GO terms were then assigned to major parental terms for biological process using the GO term Classification Counter http://www.animalgenome.org/bioinfo/tools/countgo.

For a second analysis of the expression data, control and EC_50 _level exposed samples were compared by t-tests to identify significantly changing genes. False discovery correction was not applied in the selection of the set of differential expressed genes because such approaches, while reducing type I errors for null associations can also increases the type II errors for those associations that are not null resulting in the exclusion of truly chemical responsive genes from the gene list [59,60]. Further, the potential for type I error in identifying overrepresented pathways is also controlled for by the fact that the Database for Annotation, Visualization and Integrated Discovery (DAVID) tool that was used for enrichment analysis itself includes an algorithm for multiple sample correction.

The gene list obtained from the t-test comparison was used as input into the DAVID bioinformatic resource available at http://david.abcc.ncifcrf.gov[61]. Analysis was conducted using the functional annotation clustering tool. This tool identifies annotation categories including GO terms, protein-protein interactions, protein functional domains, disease associations, bio-pathways, sequence general features, homologies, gene functional summaries, gene tissue expressions and literatures that are significantly enriched within the gene list [n.b. following multiple sample correction according to 20]. An algorithm is then used to measure the relationships between terms based on their co-associated genes, and from there to group similar, redundant and heterogeneous annotations into discrete clusters. The obtained annotation clusters can then be ranked according to the statistical significance of cluster enrichment. While pathway analysis can provide initial indications of possible effects, scrutiny of individual genes is also a useful approach that can compliment pathway analysis. Thus the expression pattern of individual annotated genes that were ascribed to significant clusters were analysed to highlight transcripts showing patterns of expression that were significantly related to exposure concentrations within a linear regression analysis. To allow open access for the community, all microarray collected was fully MIAMI compliant and has been submitted with appropriate meta-data to Gene Expression Omnibus in a MIAME complient format (as accessions GSE21008, GSE21010, GSE210011 respectively). Submission in this format allows unrestricted (open access) for future deep mining of the data.

## Authors' contributions

MB, SRS, PK, CSV, JW and DJS assisted in selection of the chemicals and strains used. SS, JW, MJJ, DJS, CS, PKH and AJM undertook all life history toxicity tests. SS, JW, SS and MJJ compiled all data relating to life-history traits and TJ conducted DEBtox modelling and undertook the mode of action analysis. JO and PK produced and fabricated all microarray and SRS, JO, JW and SRS undertook all hybridisations. MB and BAH provided all annotation information used in functional analyses. SS, DJS, MJJ, and PK undertook statistical analyses of the data-sets and DJS produced the main text and SRS, CSV, MJJ, PK and TJ contributed to the iterative refinement of the article. All authors have read and approved the submitted version.

## Supplementary Material

Additional file 1 **Fit of DEBtox models for different physiological modes of action**. Goodness of fit (as log-likelihood ratio) of different physiological DEBtox models. For each chemical, the model with the lowest log-likelihood represents the best predicted physiological mode of action. Click here for file

Additional file 2 **Assessment of micro-array sensitivity and signal linearity**. Representative analysis of the fluorescent signal generated by 10 RNAs introduced at known concentrations prior to labelling and detected by complementary reporter (8 replicates of each reporter spotted on the array). Panel A are data generated from Cadmium exposure of 40 mg/L array replicate 2, panel B is from Fluoranthene control replicate 2 and panel C is from Atrazine control replicate 6. The average signal for each of the 10 spiked transcripts is indicated by closed circles for the common reference target labelled with Cy3 whilst open squares represents the mean signal from the exposure specific target labelled with Cy5. The within array standard error bars of each measurements are shown associated with each measurement. A fitted regression line and associated R2 value is shown for the linear portion of the response for both common reference (solid line) and exposure specific target (dotted line). Click here for file

Additional file 3 **Principal Component analysis for FA excluding 0.5 mg/L**. Scores plot for PC1 and PC2 from a PCA of normalised whole genome microarray data for adult *C. elegans *exposed to a control and 4 concentrations of FA. Data from the 0.5 mg/L concentration was excluded from this analysis. Click here for file

Additional file 4 **Least squares discriminant analysis at reproduction EC_50_**. Scores plot for PC1 and PC2 from PLS-DA of normalised whole genome microarray data for adult *C. elegans *raised on control NGM media or exposed to (a) Cd, (b) FA, and (c) AZ concentrations approximating to the reproduction EC_50 _for brood size. Click here for file

## References

[B1] Barnes AI Partridge L Costing reproduction Anim Behav 2003 66 199 204 10.1006/anbe.2003.2122

[B2] Van Straalen NM Roelofs D An Introduction to Ecological Genomics 2007 Oxford, UK: Oxford University Press

[B3] Partridge L Gems D Beyond the evolutionary theory of ageing, from functional genomics to evo-gero Trends Ecol Evol 2006 21 334 340 10.1016/j.tree.2006.02.008 16769434

[B4] Filby AL Tyler CR Molecular characterization of estrogen receptors 1, 2a, and 2b and their tissue and ontogenic expression profiles in fathead minnow (*Pimephales promelas*) Biol Reprod 2005 73 648 662 10.1095/biolreprod.105.039701 15930325

[B5] Nicholson JK Wilson ID Understanding 'global' systems biology: Metabonomics and the continuum of metabolism Nat Rev Drug Discov 2003 2 668 676 10.1038/nrd1157 12904817

[B6] Quackenbush J From 'omes to biology Animal Genetics 2006 37 48 56 10.1111/j.1365-2052.2006.01476.x 16887002

[B7] Lorenz DR Cantor CR Collins JJ A network biology approach to aging in yeast Proc Natl Acad Sci USA 2009 106 1145 1150 10.1073/pnas.0812551106 19164565 PMC2629491

[B8] Zahn JM Kim SK Systems biology of aging in four species Curr Opin Biotechnol 2007 18 355 359 10.1016/j.copbio.2007.07.004 17681777 PMC3224768

[B9] Raamsdonk LM Teusink B Broadhurst D Zhang NS Hayes A Walsh MC Berden JA Brindle KM Kell DB Rowland JJ A functional genomics strategy that uses metabolome data to reveal the phenotype of silent mutations Nat Biotechnol 2001 19 45 50 10.1038/83496 11135551

[B10] Ebbels TMD Buxton BF Jones DT springScape: visualisation of microarray and contextual bioinformatic data using spring embedding and an 'information landscape' Bioinformatics 2006 22 E99 E107 10.1093/bioinformatics/btl205 16873528

[B11] Slikker W Andersen ME Bogdanffy MS Bus JS Cohen SD Conolly RB David RM Doerrer NG Dorman DC Gaylor DW Dose-dependent transitions in mechanisms of toxicity: case studies Toxicol Appl Pharmacol 2004 201 226 294 10.1016/j.taap.2004.06.027 15582646

[B12] Bundy JG Sidhu JK Rana F Spurgeon DJ Svendsen C Wren JF Stürzenbaum SR Morgan AJ Kille P Systems toxicology' approach identifies coordinated metabolic responses to copper in a terrestrial non-model invertebrate, the earthworm *Lumbricus rubellus* BMC Biol 2008 6 1 25 10.1186/1741-7007-6-25 18522721 PMC2424032

[B13] Korsloot A Van Gestel CAM Van Straalen NM Environmental Stress and Cellular Response in Arthropods 2004 London, UK: CRC Press

[B14] Kooijman SALM Dynamic Energy Budget Models in Biological Systems 2000 Cambridge: Cambridge University Press

[B15] Kooijman S Quantitative aspects of metabolic organization: a discussion of concepts Phil Trans Roy Soc B 2001 356 331 349 10.1098/rstb.2000.0771 PMC1088431 11316483

[B16] Kooijman SALM Metz JAJ On the dynamics of chemically stressed populations: the deduction of population consequences from effects on individuals Ecotox Environ Saf 1984 8 254 276 10.1016/0147-6513(84)90029-0 6734503

[B17] Kooijman S Bedaux JJM Analysis of toxicity tests on *Daphnia *survival and reproduction Water Research 1996 30 1711 1723 10.1016/0043-1354(96)00054-1

[B18] Jager T Crommentuijn T VanGestel CAM Kooijman SALM Simultaneous modeling of multiple end points in life-cycle toxicity tests Environ Sci Technol 2004 38 2894 2900 10.1021/es0352348 15212265

[B19] Jager T Alda Alvarez OA Kammenga JE Kooijman SALM Modelling nematode life cycles using dynamic energy budgets Funct Ecol 2005 19 136 144 10.1111/j.0269-8463.2005.00941.x

[B20] Benjamini Y Hochberg Y Controlling the false discovery rate - a practical and powerful approach to multiple testing J Roy Stat Soc B 1995 57 289 300

[B21] Caswell H Matrix Population Models: Construction, Analysis, and Interpretation 2001 Sunderland, MA, USA: Sinauer Associates

[B22] Anderson RM Bitterman KJ Wood JG Medvedik O Sinclair DA Nicotinamide and PNC1 govern lifespan extension by calorie restriction in *Saccharomyces cerevisiae* Nature 2003 423 181 185 10.1038/nature01578 12736687 PMC4802858

[B23] Lee SS Lee RYN Fraser AG Kamath RS Ahringer J Ruvkun G A systematic RNAi screen identifies a critical role for mitochondria in *C. elegans *longevity Nat Genet 2003 33 40 48 10.1038/ng1056 12447374

[B24] Wood JG Rogina B Lavu S Howitz K Helfand SL Tatar M Sinclair D Sirtuin activators mimic caloric restriction and delay ageing in metazoans Nature 2004 430 686 689 10.1038/nature02789 15254550

[B25] Murphy CT McCarroll SA Bargmann CI Fraser A Kamath RS Ahringer J Li H Kenyon C Genes that act downstream of DAF-16 to influence the lifespan of *Caenorhabditis elegans* Nature 2003 424 277 284 10.1038/nature01789 12845331

[B26] Hsu AL Murphy CT Kenyon C Regulation of aging and age-related disease by DAF-16 and heat- shock factor Science 2003 300 1142 1145 10.1126/science.1083701 12750521

[B27] Cheng Q Valmas N Reilly PEB Collins PJ Kopittke R Ebert PR *Caenorhabditis elegans *mutants resistant to phosphine toxicity show increased longevity and cross-resistance to the synergistic action of oxygen Toxicol Sci 2003 73 60 65 10.1093/toxsci/kfg049 12700416

[B28] Zheng X Yang Z Yue Z Alvarez JD Sehgal A FOXO and insulin signaling regulate sensitivity of the circadian clock to oxidative stress Proc Natl Acad Sci USA 2007 104 15899 15904 10.1073/pnas.0701599104 17895391 PMC2000406

[B29] Baumeister R Schaffitzel E Hertweck M Endocrine signaling in *Caenorhabditis elegans *controls stress response and longevity J Endocrinol 2006 190 191 202 10.1677/joe.1.06856 16899554

[B30] Lamitina ST Strange K Transcriptional targets of DAF-16 insulin signaling pathway protect *C. elegans *from extreme hypertonic stress Am J Physiol Cell Physiol 2005 288 C467 C474 10.1152/ajpcell.00451.2004 15496475

[B31] McElwee JJ Schuster E Blanc E Thomas JH Gems D Shared transcriptional signature in *Caenorhabditis elegans *dauer larvae and long-lived daf-2 mutants implicates detoxification system in longevity assurance J Biol Chem 2004 279 44533 44543 10.1074/jbc.M406207200 15308663

[B32] Kooijman SALM Bedaux JJM Analysis of toxicity tests on fish growth Water Research 1996 30 1633 1644 10.1016/0043-1354(96)00057-7

[B33] Alda Alvarez OA Jager T Kooijman SALM Kammenga JE Responses to stress of *Caenorhabditis elegans *populations with different reproductive strategies Funct Ecol 2005 19 656 664 10.1111/j.1365-2435.2005.01012.x

[B34] Alda Alvarez OA Jager T Redondo EM Kammenga JE Physiological modes of action of toxic chemicals in the nematode *Acrobeloides nanus* Environ Toxicol Chem 2006 25 3230 3237 10.1897/06-097R.1 17220093

[B35] Jager T Heugens EHW Kooijman SALM Making sense of ecotoxicological test results: Towards application of process-based models Ecotoxicology 2006 15 305 314 10.1007/s10646-006-0060-x 16739032

[B36] Jones D Candido EPM Feeding is inhibited by sublethal concentrations of toxicants and by heat stress in the nematode *Caenorhabditis elegans*: Relationship to the cellular stress response J Exp Zool 1999 284 147 157 10.1002/(SICI)1097-010X(19990701)284:2<147::AID-JEZ4>3.0.CO;2-Z 10404644

[B37] Alda Alvarez OA Jager T Colao BN Kammenga JE Temporal dynamics of effect concentrations Environ Sci Technol 2006 40 2478 2484 10.1021/es052260s 16646492

[B38] Reichert K Menzel R Expression profiling of five different xenobiotics using a *Caenorhabditis elegans *whole genome microarray Chemosphere 2005 61 229 237 10.1016/j.chemosphere.2005.01.077 16168746

[B39] Cui YX McBride SJ Boyd WA Alper S Freedman JH Toxicogenomic analysis of *Caenorhabditis elegans *reveals novel genes and pathways involved in the resistance to cadmium toxicity Genome Biology 2007 8 R122 10.1186/gb-2007-8-6-r122 17592649 PMC2394766

[B40] Menzel R Yeo HL Rienau S Li S Steinberg CEW Sturzenbaum SR Cytochrome P450s and short-chain dehydrogenases mediate the toxicogenomic response of PCB52 in the nematode *Caenorhabditis elegans* J Mol Biol 2007 370 1 13 10.1016/j.jmb.2007.04.058 17499272

[B41] Menzel R Sturzenbaum S Barenwaldt A Kulas J Steinberg CEW Humic material induces behavioral and global transcriptional responses in the nematode *Caenorhabditis elegans* Environ Sci Technol 2005 39 8324 8332 10.1021/es050884s 16294870

[B42] Jain PT Chang SH Gutry PP Berezesky IK Trump BF The relationship between [CA2+] and cell-death using and in-vivo model - a study using the ced-1 mutant strain of *C. elegans* Toxicol Pathol 1993 21 572 583 10.1177/019262339302100608 8052804

[B43] Wang SC Tang ML Pei B Xiao X Wang J Hang HY Wu LJ Cadmium-induced germline apoptosis in *Caenorhabditis elegans*: The roles of HUS1, p53, and MAPK signaling pathways Toxicol Sci 2008 102 345 351 10.1093/toxsci/kfm220 17728284

[B44] Roh JY Jung IH Lee JY Choi JH Toxic effects of di(2-ethylhexyl)phthalate on mortality, growth, reproduction and stress-related gene expression in the soil nematode *Caenorhabditis elegans* Toxicology 2007 237 126 133 10.1016/j.tox.2007.05.008 17604895

[B45] Jin YH Dunlap PE McBride SJ Al-Refai H Bushel PR Freedman JH Global transcriptome and deletome profiles of yeast exposed to transition metals PLOS Genetics 2008 4 e1000053 10.1371/journal.pgen.1000053 18437200 PMC2278374

[B46] Causton HC Ren B Koh SS Harbison CT Kanin E Jennings EG Lee TI True HL Lander ES Young RA Remodeling of yeast genome expression in response to environmental changes Mol Biol Cell 2001 12 323 337 11179418 10.1091/mbc.12.2.323 PMC30946

[B47] Chen DR Toone WM Mata J Lyne R Burns G Kivinen K Brazma A Jones N Bahler J Global transcriptional responses of fission yeast to environmental stress Mol Biol Cell 2003 14 214 229 10.1091/mbc.E02-08-0499 12529438 PMC140239

[B48] Swain SC Keusekotten K Baumeister R Sturzenbaum SR *C. elegans *metallothioneins: New insights into the phenotypic effects of cadmium toxicosis J Mol Biol 2004 341 951 959 10.1016/j.jmb.2004.06.050 15328611

[B49] Dallinger R Berger B Gruber C Hunziker P Sturzenbaum S Metallothioneins in terrestrial invertebrates: Structural aspects, biological significance and implications for their use as biomarkers Cell Mol Biol 2000 46 331 346 10774923

[B50] Owen J Hedley BA Svendsen C Wren J Jonker MJ Hankard PK Lister LJ Stürzenbaum SR Morgan AJ Spurgeon DJ Transcriptome profiling of developmental and xenobiotic responses in a keystone soil animal, the oligochaete annelid *Lumbricus rubellus* BMC Genomics 2008 6 266 10.1186/1471-2164-9-266 PMC2440553 18522720

[B51] Filipic M Fatur T Vudrag M Molecular mechanisms of cadmium induced mutagenicity Hum Exp Toxicol 2006 25 67 77 10.1191/0960327106ht590oa 16539211

[B52] Ibiam U Grant A RNA/DNA ratios as a sublethal endpoint for large-scale toxicity tests with the nematode *Caenorhabditis elegans* Environ Toxicol Chem 2005 24 1155 1159 10.1897/04-262R.1 16110994

[B53] Jones OAH Spurgeon DJ Svendsen C Griffin JL A metabolomics based approach to assessing the toxicity of the polyaromatic hydrocarbon pyrene to the earthworm *Lumbricus rubellus* Chemosphere 2008 71 601 609 10.1016/j.chemosphere.2007.08.056 17928029

[B54] Connor SC Wu W Sweatman BC Manini J Haselden JN Crowther DJ Waterfield CJ Effects of feeding and body weight loss on the H-1-NMR-based urine metabolic profiles of male Wistar Han rats: implications for biomarker discovery Biomarkers 2004 9 156 179 10.1080/13547500400006005 15370873

[B55] Park SK Tedesco PM Johnson TE Oxidative stress and longevity in *Caenorhabditis elegans *as mediated by SKN-1 Aging Cell 2009 8 258 269 10.1111/j.1474-9726.2009.00473.x 19627265 PMC2762118

[B56] Garaj-Vrhovac V Zeljezic D Assessment of genome damage in a population of Croatian workers employed in pesticide production by chromosomal aberration analysis, micronucleus assay and Comet assay J Appl Toxicol 2002 22 249 255 10.1002/jat.855 12210542

[B57] Tennant AH Peng BC Kligerman AD Genotoxicity studies of three triazine herbicides: in vivo studies using the alkaline single cell gel (SCG) assay Mutation Research-Genetic Toxicology and Environmental Mutagenesis 2001 493 1 10 10.1016/S1383-5718(01)00145-0 11516710

[B58] Altenburger R Nendza M Schuurmann G Mixture toxicity and its modeling by quantitative structure-activity relationships Environ Toxicol Chem 2003 22 1900 1915 10.1897/01-386 12924589

[B59] Perneger TV What's wrong with Bonferroni adjustments Br Med J 1998 316 1236 1238 10.1136/bmj.316.7139.1236 PMC1112991 9553006

[B60] Rothman KJ No adjustments are needed for multiple comparisons Epidemiology 1990 1 43 46 2081237

[B61] Huang DW Sherman BT Tan Q Kir J Liu D Bryant D Guo Y Stephens R Baseler MW Lane HC Lempicki RA DAVID Bioinformatics Resources: expanded annotation database and novel algorithms to better extract biology from large gene lists Nucleic Acid Research 2007 35 W169 175 10.1093/nar/gkm415 PMC1933169 17576678

