# Supplementary figures and images for: Linking toxicant physiological mode of action with induced gene expression changes in Caenorhabditis elegans
Source: BMC Syst Biol. 2010 Mar 23;4:32. doi: 10.1186/1752-0509-4-32 (PMC2857823; doi:10.1186/1752-0509-4-32)

## Slide 1
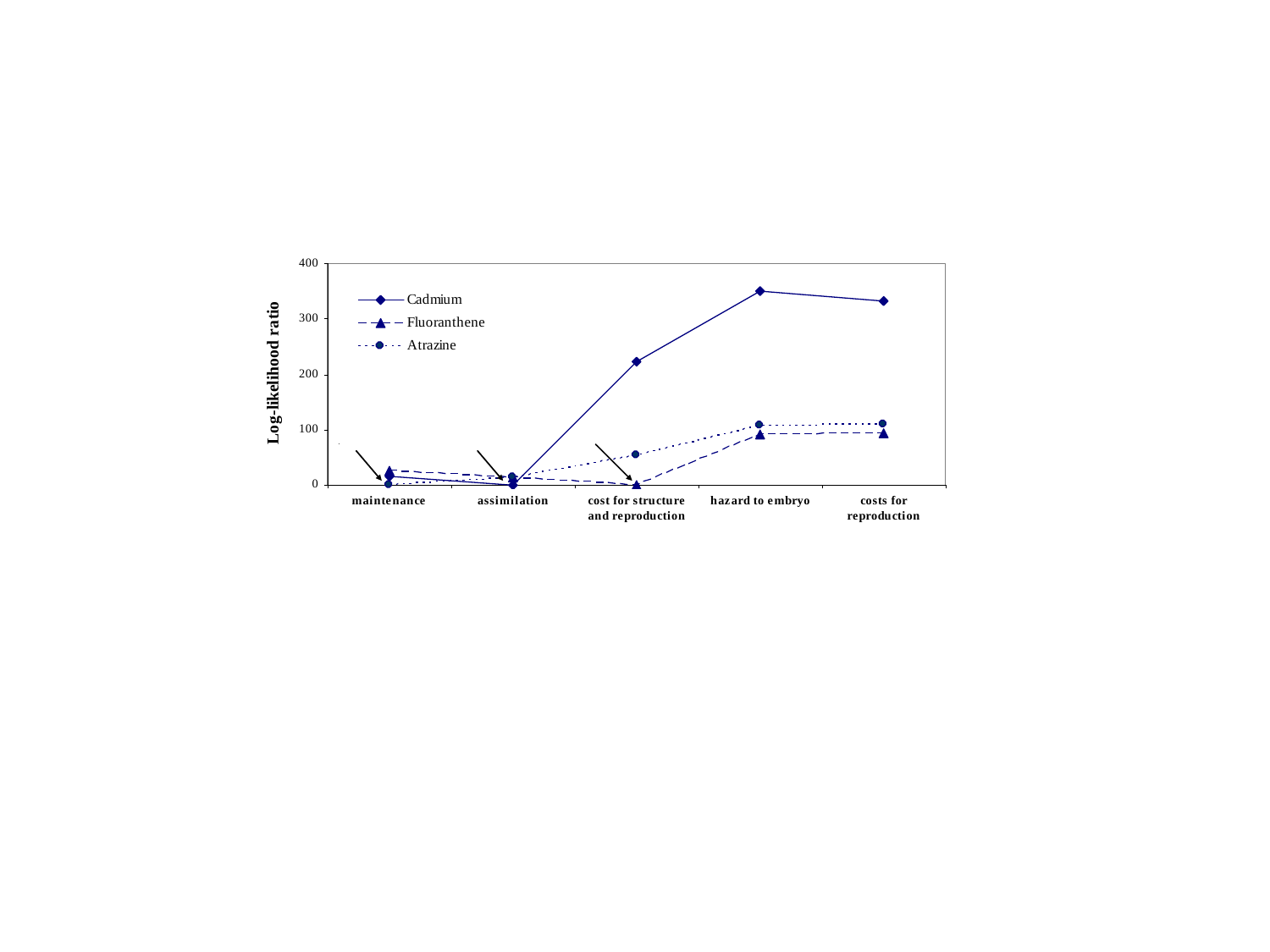

Supplement: Additional file 1 — Fit of DEBtox models for different physiological modes of action. Goodness of fit (as log-likelihood ratio) of different physiological DEBtox models. For each chemical, the model with the lowest log-likelihood represents the best predicted physiological mode of action. [file 1752-0509-4-32-S1.PPT]

## Slide 1
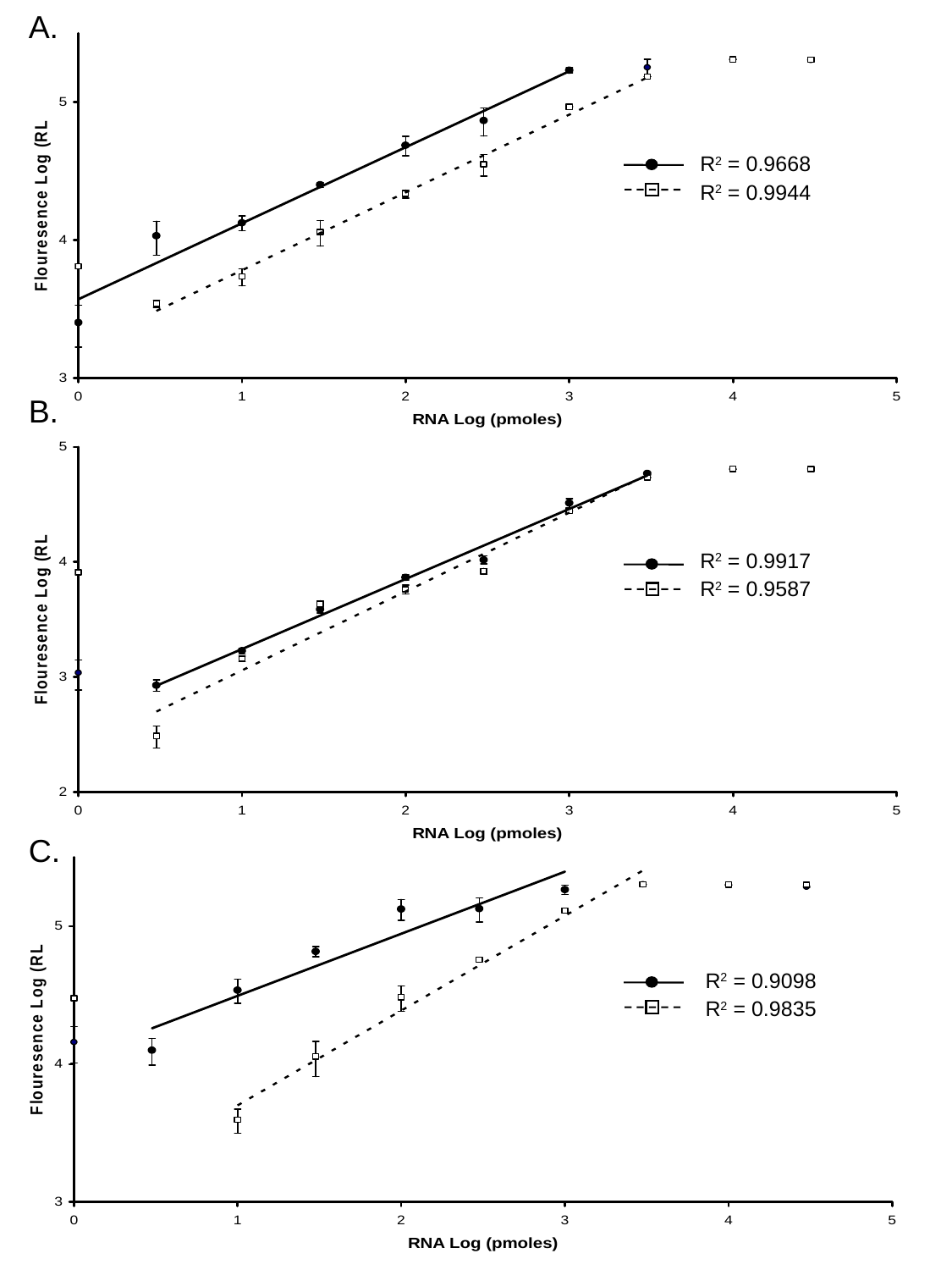

A.
R2 = 0.9668
R2 = 0.9944
B.
R2 = 0.9917
R2 = 0.9587
C.
R2 = 0.9098
R2 = 0.9835

Supplement: Additional file 2 — Assessment of micro-array sensitivity and signal linearity. Representative analysis of the fluorescent signal generated by 10 RNAs introduced at known concentrations prior to labelling and detected by complementary reporter (8 replicates of each reporter spotted on the array). Panel A are data generated from Cadmium exposure of 40 mg/L array replicate 2, panel B is from Fluoranthene control replicate 2 and panel C is from Atrazine control replicate 6. The average signal for each of the 10 spiked transcripts is indicated by closed circles for the common reference target labelled with Cy3 whilst open squares represents the mean signal from the exposure specific target labelled with Cy5. The within array standard error bars of each measurements are shown associated with each measurement. A fitted regression line and associated R2 value is shown for the linear portion of the response for both common reference (solid line) and exposure specific target (dotted line). [file 1752-0509-4-32-S2.PPT]

## Slide 1
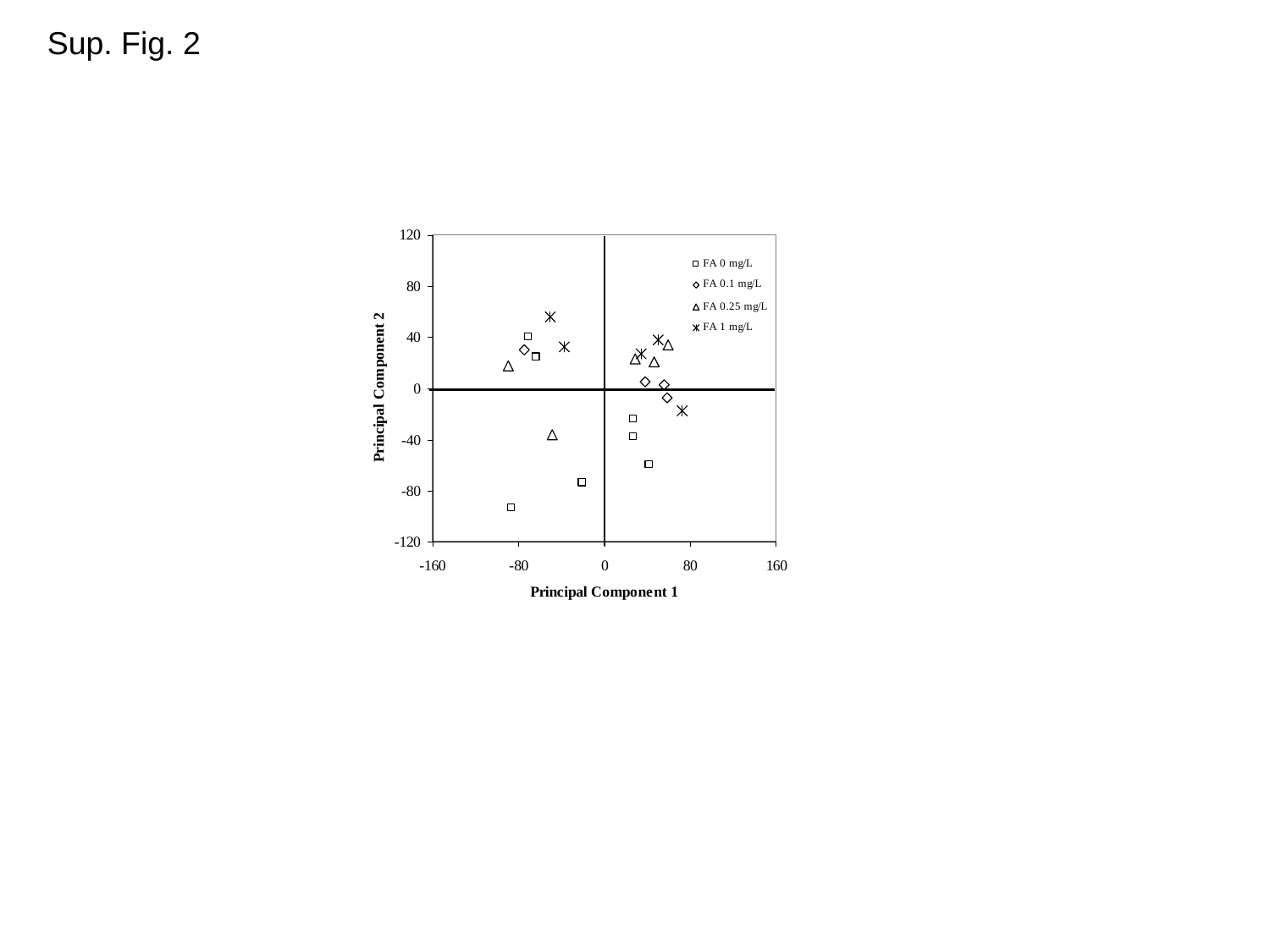

Sup. Fig. 2

Supplement: Additional file 3 — Principal Component analysis for FA excluding 0.5 mg/L. Scores plot for PC1 and PC2 from a PCA of normalised whole genome microarray data for adult C. elegans exposed to a control and 4 concentrations of FA. Data from the 0.5 mg/L concentration was excluded from this analysis. [file 1752-0509-4-32-S3.PPT]

## Slide 1
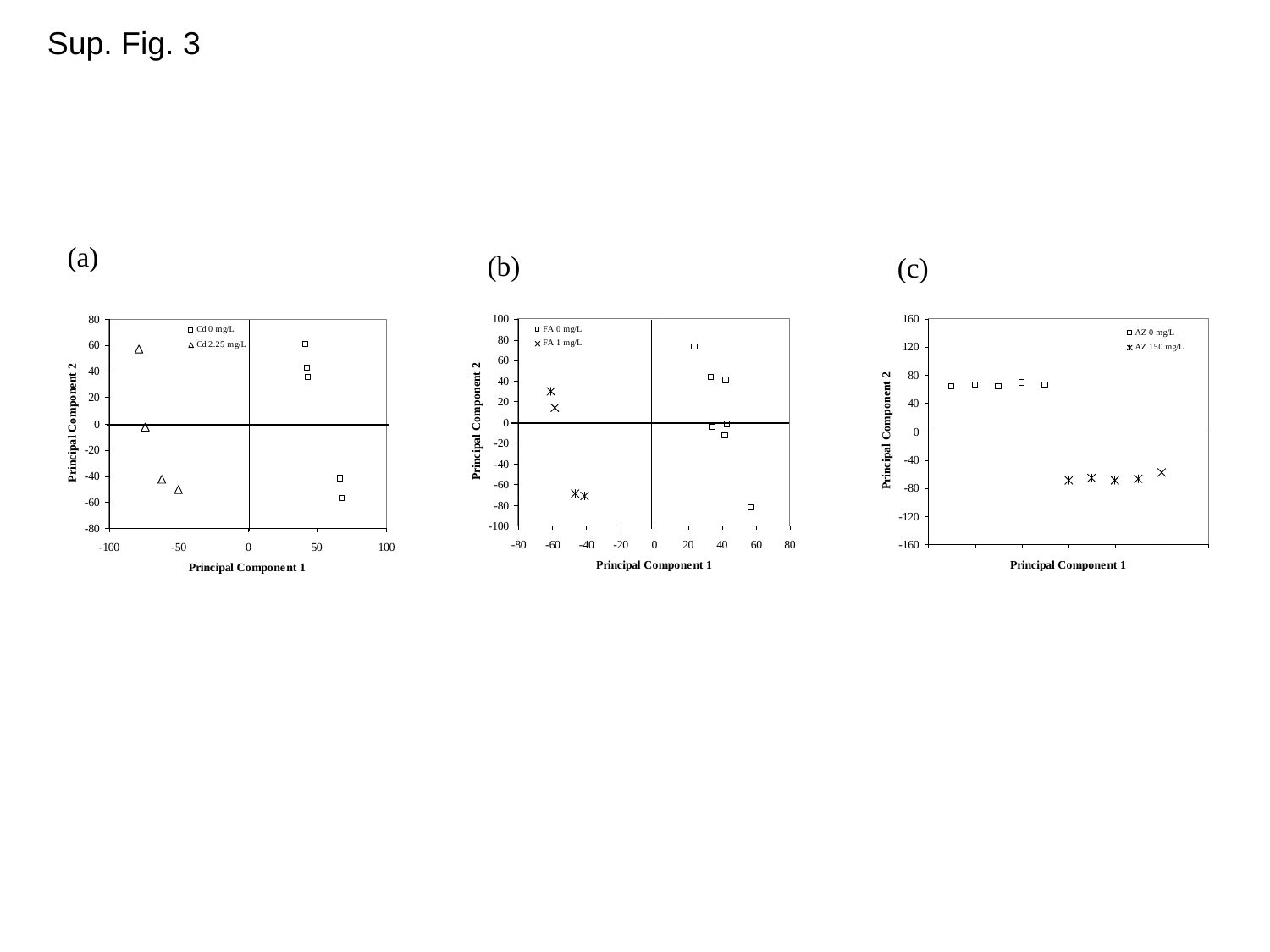

Sup. Fig. 3
(a)
(b)
(c)

Supplement: Additional file 4 — Least squares discriminant analysis at reproduction EC50. Scores plot for PC1 and PC2 from PLS-DA of normalised whole genome microarray data for adult C. elegans raised on control NGM media or exposed to (a) Cd, (b) FA, and (c) AZ concentrations approximating to the reproduction EC50 for brood size. [file 1752-0509-4-32-S4.PPT]
